# Genome-Wide Characterization and Expression Analyses of *Pleurotus ostreatus* MYB Transcription Factors during Developmental Stages and under Heat Stress Based on de novo Sequenced Genome

**DOI:** 10.3390/ijms19072052

**Published:** 2018-07-14

**Authors:** Lining Wang, Wei Gao, Xiangli Wu, Mengran Zhao, Jibin Qu, Chenyang Huang, Jinxia Zhang

**Affiliations:** 1 Institute of Agricultural Resources and Regional Planning, Chinese Academy of Agricultural Sciences, Beijing 100081, China; wanglining90@126.com (L.W.); gaowei01@caas.cn (W.G.); wuxiangli@caas.cn (X.W.); zhaomengran@caas.cn (M.Z.); qujibin@caas.cn (J.Q.); huangchenyang@caas.cn (C.H.); 2 Key Laboratory of Microbial Resources, Ministry of Agriculture, Beijing 100081, China

**Keywords:** *Pleurotus ostreatus*, de novo, NGS, Bionano, MYB, development, heat stress

## Abstract

*Pleurotus ostreatus* is a commercially grown mushroom species in China. However, studies on the mechanisms of the fruiting body development and stress response of *P. ostreatus* are still at a primary stage. In this study, we report the entire genome sequence of *P. ostreatus* CCMSSC03989. Then, we performed comprehensive genome-wide characterization and expression analysis of the MYB transcription factor family during a series of developmental stages and under the condition of heat stress. A 34.76 Mb genome was obtained through next-generation sequencing (NGS) and Bionano optical mapping approaches. The genome has a scaffold N50 of 1.1 Mb and contains 10.11% repeats, and 10,936 gene models were predicted. A total of 20 *MYB* genes (*PoMYB*) were identified across the genome, and the full-length open reading frames were isolated. The *PoMYBs* were classified into 1 repeat (1R), 2R, and 3R-MYB groups according to their MYB domain repeat numbers, and 3R-MYBs possessed relatively more introns than 1R and 2R-MYBs. Based on phylogenetic analysis, the *PoMYBs* were divided into four groups and showed close relationships with the *MYB* genes of plants and fungi. RNA-sequencing (RNA-Seq) and quantitative PCR (qPCR) analyses revealed that *PoMYB* expression showed stage-specific patterns in reproductive stages and could be induced by heat stress. The *P. ostreatus* draft genome will promote genome-wide analysis, and our study of *PoMYBs* will promote further functional analysis of *MYB* genes in mushrooms.

## 1. Introduction

*Pleurotus ostreatus* is widely cultivated in China for its delicious taste, nutritional value [1], ease of growth on a variety of organic substrates [2], and tolerance to a wide temperature range during cultivation [3]. In China, *P. ostreatus* is mostly cultivated under horticultural facilities and has a yield of 5.38 million tons in 2016, which is much higher than the yield of globally cultivated *Agaricus bisporus* (http://mushroomsci.org/html/001/abfe8d1b-b.html). Genome sequences are valuable resources for genetic or molecular-based analysis, which is beneficial to the decryption of genetic diversity and genes controlling important agronomic traits [4]. To date, two genomes of *P. ostreatus*, PC15 [5] and CCEF00389 [6], are available, and the PC15 genome has been widely used since its release [7,8]. Several commercial strains are used in production at present [9], and they are genetically diverse according to identification by using molecular markers [10]. Thus, the genomes of PC15 and CCEF00389 do not sufficiently represent the genetic diversity of *P. ostreatus*. Obtaining the genome sequences of more strains for various research purposes is thus necessary. So far, the genomes of several edible mushrooms, including *P. tuoliensis* [11], *Volvariella volvacea* [12], *Flammulina velutipes* [13,14], and *Lentinula edodes* [15], have been sequenced. Genome-wide study of mushrooms has been conducted in a variety of aspects; that is, identifying fruiting body formation-related genes [14], studying the molecular mechanism of lignocellulose degradation [13,15], identifying and characterizing small noncoding RNAs [6], mining microsatellites [16], and so forth.

The mushroom fruiting body is the most conspicuous structure in fungi, and its formation represents a highly complex developmental progress. In China, *P. ostreatus* is mostly cultivated under horticultural facilities, and high temperature is one of the most common environmental factors that negatively affects the yield and quality of the fruiting body. Recently, some functional genes and signaling pathways related to mushroom development have been identified and characterized [17,18,19,20]. Studies on heat stress mechanisms in mushrooms have mainly focused on carbon metabolism (trehalose) [21,22], antioxidant enzymes [23,24], nitric oxide [25,26], and morphological features [27]. In addition, transcription factors are widely involved in both fungal development [28,29] and heat stress responses [30]. Among the most studied transcription factors, the *MYB* gene family comprises one of the largest transcription factors and is widely distributed in eukaryotes [31,32,33,34].

The *MYB* genes of plants play diverse roles in organ development, cell shape determination [31], secondary metabolism [35], and abiotic stress tolerance [36,37]. Similarly, previous studies have proven that *MYB* genes are involved or required in several processes in fungi, including spore development [33,38], sexual development [34,39,40], metabolism [41], and abiotic stress response [42]. To date, most genome wide analyses of *MYB* genes focused on plant species, such as *Arabidopsis* [43], *Oryza sativa* subsp. *Indica* [32], *Glycine max* [44], *Gossypium hirsutum* [45], and Chinese cabbage [46]. Studies on transcription factors in mushrooms are limited compared to those of plants [47], and studies on *MYBs* are even fewer in number.

MYB transcription factors have one to four imperfect MYB repeats, each of which contains approximately 50 amino acids [48]. Each repeat gives rise to a helix–turn–helix (HTH) secondary structure that binds directly to the major groove of DNA, and several conserved and regularly spaced tryptophans in each repeat form a hydrophobic core to maintain the HTH structure [49]. According to the repeat numbers that the MYB domain contains, the *MYB* gene family can be classified into the following four subfamilies: 4R-MYB, 3R-MYB, 2R-MYB, and 1R-MYB (MYB domain containing a single repeat or partial MYB repeat; also known as *MYB*-related genes) [31].

In this study, we report a draft genome sequence of the monokaryotic *P. ostreatus* strain CCMSSC03989-1 (China Center for Mushroom Spawn Standards and Control, Beijing, China), based on the combination approaches of NGS and optical maps, and we conducted a comprehensive genome-wide analysis of the *MYB* gene family based on the obtained genome sequence. A total of 20 *MYB* genes were identified and subsequently subjected to gene structure analysis, phylogenetic analysis, and transcriptome (RNA-Seq) and qPCR analysis during developmental stages and under heat stress. The genome-wide analysis of MYB members might contribute to future studies on the functional characterization of MYB proteins in *P. ostreatus*. Findings of this study will provide a solid foundation to determine the molecular and regulatory mechanisms of MYB transcription factors during the development and under heat stress of mushrooms.

## 2. Results

### 2.1. Genome Sequencing, Assembly, and Annotation

**Figure 1:**
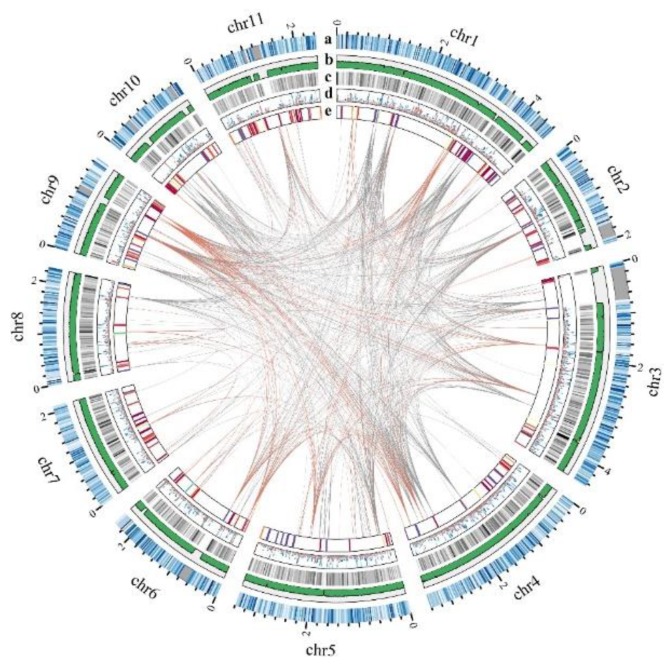
Global view of the *P. ostreatus* genome. Track **a** denotes the 11 pseudo-chromosomes of *P. ostreatus* (Mb). The positions of the Nt.BspQI labels in the optical map are shown as vertical blue lines (the deeper the color, the greater the density) and gaps are shown as gray blocks. Track **b** shows the percentage of G + C in 20 kb non-overlapping windows. Track **c** shows the gene density in 20 kb non-overlapping windows. Track **d** shows the gene expression of control (gray) and heat stress (orange) condition; mycelia (M), primordia (P), fruiting bodies (F), and spores (S) (M, P, F, S: from light blue to deep blue). Track **e** shows large segmental duplications with orange (sequence length ≥ 5 kb) and gray (sequence length ≥ 2 kb).

We sequenced the haploid of *P. ostreatus* strain CCMSSC03989-1 using a whole-genome shotgun and mate-pair sequencing strategy, and the NGS assembly was hybrid-scaffolded by using Bionano optical maps. A genome sequence of 36.42 Mb was obtained by assembling approximately 53.72 million clean reads (~341X coverage) (Table S1). This genome sequence assembly consisted of 825 scaffolds with a contig N50 of 126.35 kb and a scaffold N50 of 432.52 kb. To achieve a better assembly of the *P. ostreatus* genome, a high-throughput whole-genome mapping technique by nanochannel in a Bionano Genomics Irys system was employed. We obtained a total of 7.05 Gb clean data, which were assembled to 246 maps from the Irys system (Table S2). Combining the NGS scaffolds and Bionano maps, the hybrid scaffolding produced a 34.76 Mb genome with higher quality (Figure S1): 203 scaffolds with a contig N50 of 191.92 kb and a scaffold N50 of 1109.40 kb (Table 1). The final scaffold N50 had a 2.56X improvement, which indicated that the genome map is capable of assisting genome assembly with NGS data. Furthermore, the K-mer Analysis Toolkit (KAT) and Benchmarking Universal Single-Copy Orthologs (BUSCOs) were used for assembly quality assessment. In the KAT histograms, K-mers that occurred once in the assembly showed a perfect peak and K-mers not occurring in the assembly did not show any peak, which represented high completeness of the assembly (Figure S2); a total of 278 (95.8% of all the fungi odb9) BUSCOs were determined in this assembly, of which five BUSCOs were fragments and 273 BUSCOs were fully annotated (Table S3). The above analysis confirmed that the assembled genome of *P. ostreatus* had high completeness and accuracy. Using the Bionano HybridScaffolding pipeline, 69 scaffolds (84.88% of the full length) were anchored to the 11 chromosomes of PC15 (Figure 1, Table S4). Although the whole genome sequences of CCMSSC03989 and PC15 showed an extremely high level of sequence colinearity (Figure S3), considerable structural variations were found between them (Table S5).

Repetitive sequences represent approximately 10.11% of the genome. The majority of the repeats is comprised by long terminal repeats (LTR, 4.28% of the genome; Table S6). The G + C content of the assembly is 50.49%. In total, 10,936 protein-coding gene models were predicted with an average sequence length of 1743 bp. Approximately 59.30% of the annotations can be assigned to gene ontology (GO) catalogs, 95.92% can be assigned to Non-Redundant protein sequences (NR), 88.51% can be assigned to InterProScan, and 36.10% can be assigned to Kyoto Encyclopedia of Genes and Genomes (KEGG) pathways.

**Figure 2:**
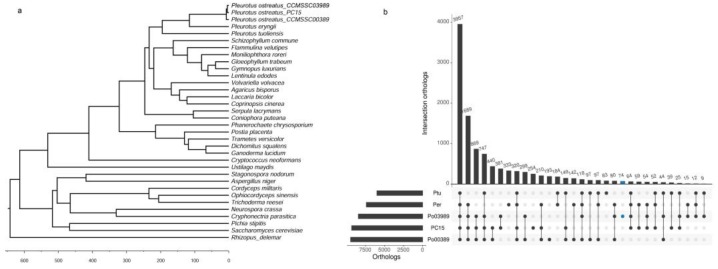
Orthologous groups and phylogenetic tree of *P. ostreatus* and other fungal species. (**a**) Phylogenetic tree and divergence data of 34 species based on the proteins of 131 single-copy genes annotated to the genome sequence of each species; (**b**) Distribution of orthologous groups in Ptu (*P. tuoliensis*), Per (*P. eryngii*), Po03989 (*P. ostreatus*_CCMSSC03989), PC15 (*P. ostreatus*_PC15), and Po00389 (*P. ostreatus*_CCEF00389).

Ortholog analysis was conducted together with the other 33 fungus species (Table S7). OrthoMCL analysis revealed that *P. ostreatus* was assigned into 8437 orthologous groups and 44 groups (153 proteins) are unique to *P. ostreatus*. In addition, 3957 orthologous groups were identified among the three *Pleurotus* species; that is, *P. ostreatus*, *P. tuoliensis*, and *P. eryngii* (Figure 2b). A total of 131 single-copy orthologous genes were identified by ortholog analysis and were sequentially used for constructing a phylogenetic tree (Figure 2a) based on the maximum likelihood (ML) method. The topology of the tree was consistent with the taxonomic classification of these species. PC15 had the closest evolutionary affinity with CCMSSC03989 among all the compared species, diverging approximately 4.48 million years ago.

### 2.2. Identification and Classification of MYB Genes in P. ostreatus

**Figure 3:**
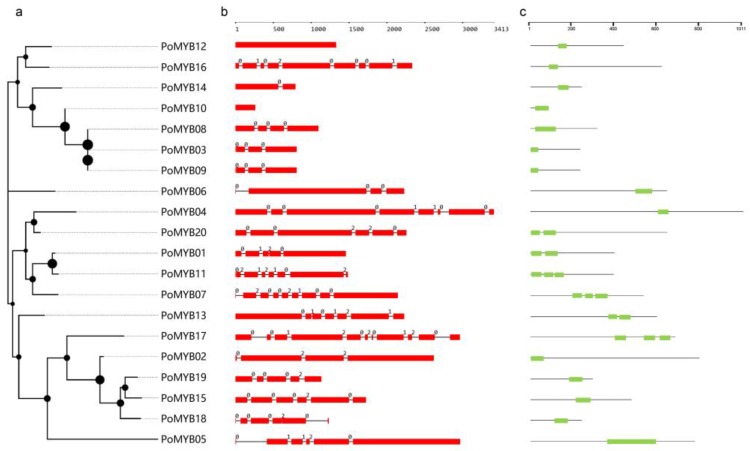
Phylogenetic relationship, gene structure, and MYB domain of *PoMYBs*. (**a**) A maximum likelihood (ML) phylogenetic tree with bootstrap 1000 based on the MYB domains; the dots on the nodes represent bootstrap values; (**b**) Gene structures of *PoMYBs*: the exons are represented by red rectangles, the black lines connecting two exons represent introns, and the numbers above the line represent the intron phase; (**c**) MYB domain distribution in *PoMYBs*, the green rectangles represent MYB domains.

In total, 16, 16, and 24 candidate *MYB* genes were predicted from the PC15, CCEF00389, and CCMSSC03989 genome, respectively. Among the 24 *MYB* genes of CCMSSC03989, the full-length open reading frames of 20 genes were successfully amplified. These 20 genes were named from *PoMYB01* to *PoMYB20* and were used for the following study (Table S8). The sequences were submitted and assigned the GenBank accession numbers MH510308–MH510327 (Table S8). Of these *PoMYBs*, *PoMYB09* was identified as a duplication of *PoMYB03*, and these two *PoMYBs* were located in different pseudo-chromosomes. All the *MYB* genes contained one to three MYB or MYB-like repeats. Based on the number of repeats, we classified the 20 *PoMYBs* into three groups: namely, 1R-MYB, 2R-MYB, and 3R-MYB (Figure 3c). *PoMYB07*, *PoMYB11*, and *PoMYB17* belong to the 3R-MYB; *PoMYB01*, *PoMYB13*, and *PoMYB20* to the 2R-MYB; and the other 14 *PoMYBs* to the 1R-MYB.

### 2.3. Sequence Analysis, Gene Structure, and Phylogenetic Analysis of PoMYBs

The gene length of *PoMYBs* varied from 261 to 3033 bp, and the encoded protein length was 87–1011 amino acids, with an average of 486 amino acids. The average molecular weight was approximately 53.77 kilodaltons (kDa), and the mean isoelectric point (pI) value was 6.85 (Table S8). To understand the structural component of *PoMYBs*, we obtained their exon and intron organizations by comparing the complementary DNA (cDNA) sequences with the corresponding genomic DNA sequences (Figure 3b). The gene structures were variable among the 20 *PoMYBs*, except for the fact that all the multiple-exon *PoMYBs* began with a phase-zero intron. Two 1R-MYB genes, *PoMYB10* and *PoMYB12*, are intronless genes; the 3R-MYB gene, *PoMYB17*, had a maximal number of introns of 10. On average, the 1R-MYBs were disrupted by 3.6 introns, 2R-MYBs were disrupted by 5 introns, and 3R-MYBs were disrupted by 8.3 introns.

Analyses revealed that the MYB domains of *P. ostreatus* were divergently distributed across the gene not only in the N-terminal, but also in the middle or C-terminal regions of the proteins, reflecting the high sequence divergence (Figure 3c). On the basis of the MYB domain sequences, a ML tree was built. In the ML tree, the 20 *PoMYBs* were clustered into four groups (Figure 3a). *PoMYB06* was clustered into a single-branch clade, indicating its independent origin. 2R-MYBs and 3R-MYBs showed a close relationship, which might be attributed to the origin of 2R-MYB proteins from 3R-MYB proteins because of the loss of one repeat [50]. Furthermore, an unrooted ML phylogenetic tree was constructed by using 104 *MYB* genes of other organisms (including fungi, plants, and animals) (Figure S4, Table S9), and 20 *PoMYBs* were classified into 14 clades. As shown in this ML tree, most *PoMYBs* showed high similarity with those of plants or fungi, except for *PoMYB06*, which exhibited high similarity with animals.

### 2.4. Expression of PoMYBs during Different Developmental Stages and under Heat Stress of P. ostreatus

**Figure 4:**
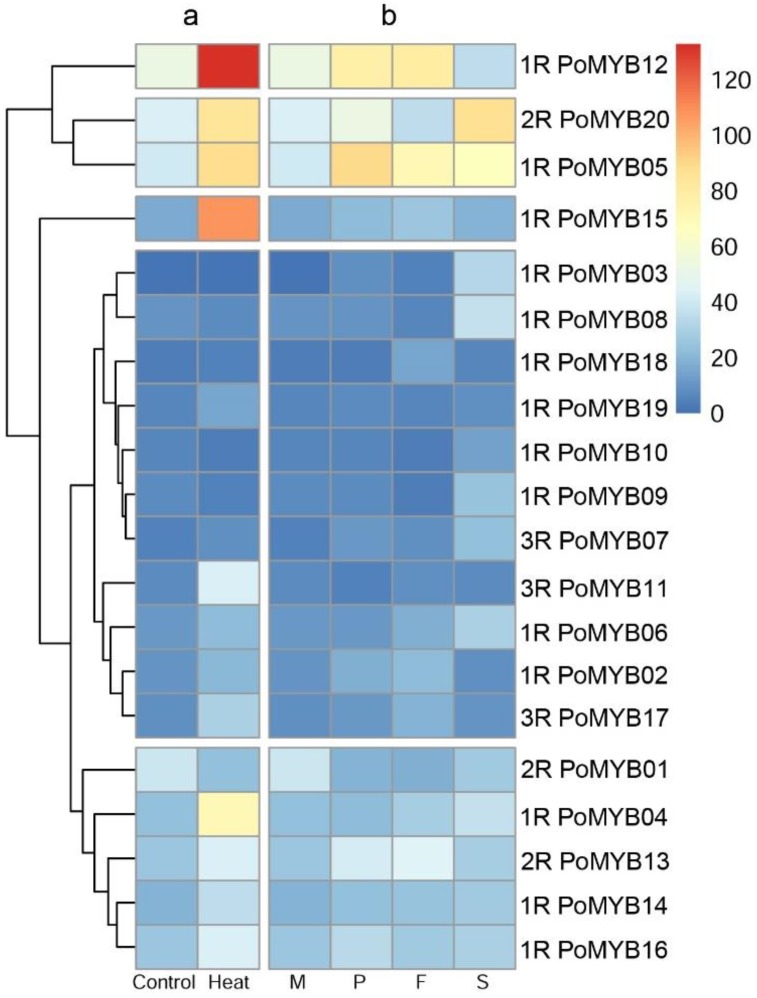
Heatmap of the 20 *PoMYBs* in different developmental stages and under heat stress. (**a**) RNA-Seq analysis of *PoMYBs* under heat stress; (**b**) RNA-Seq analysis of the *PoMYBs* during developmental stages. M = mycelia (the same as Control); P = primordia; F = fruiting bodies; S = spores. Genes highly or weakly expressed are colored red and blue, respectively. 1R, 2R, and 3R represent 1R-MYB, 2R-MYB, and 3R-MYB, respectively. The heatmap was generated using the pheatmap package in R, and the color scale shown at the top represents FPKM (fragments per kilobase of exon per million mapped reads) counts.

**Figure 5:**
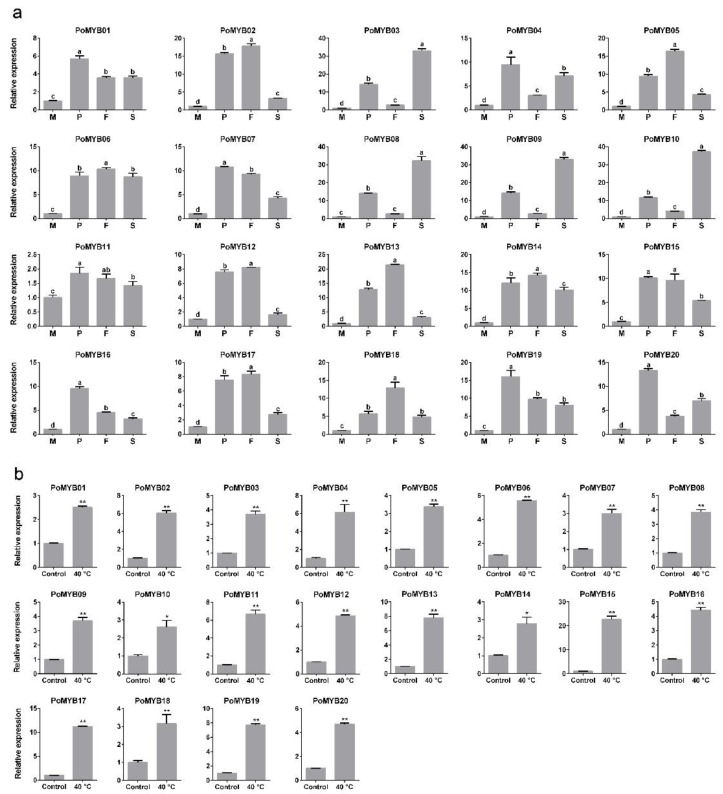
The expression analysis of 20 *PoMYBs* using qPCR. (**a**) Differential expression of *PoMYBs* during developmental stages. M = mycelia (the same as Control); P = primordia; F = fruiting bodies; S = spores. The different letters over the columns denote significant differences (*p <* 0.05); (**b**) Differential expression of *PoMYBs* under heat stress. Gene expression levels are presented relative to that in mycelia. Statistical analysis was performed using the *t* test. * *p* < 0.05, ** *p* < 0.01. Mean values and standard deviations of three biological replicates are shown.

As revealed by the RNA-Seq (Figure 4b), all 3R-MYBs showed a low level of transcript abundance during the whole developmental stage, 2R-MYBs showed a relatively high level of transcript abundance, and 1R-MYBs showed a wide range of transcript abundance. Nineteen *PoMYBs* exhibited upregulated expression in at least one reproductive stage, which was supported by the data of both RNA-Seq (Figure 4b) and qPCR (Figure 5a). Among them, 11 *PoMYBs* were upregulated during all the reproductive stages, indicating their extensive roles during the development of *P. ostreatus*. Specifically, *PoMYB16* showed maximal expression at the stage of primordia; *PoMYB02*, *PoMYB12*, *PoMYB13*, *PoMYB17*, and *PoMYB18* showed maximal expression in fruiting bodies; *PoMYB03*, *PoMYB08*, *PoMYB09*, and *PoMYB10* showed maximal expression in spores (Figure 4b and Figure 5a). These results indicated that *PoMYB* genes may play stage-specific regulatory roles during the development of *P. ostreatus.* The stage-specific expression patterns might facilitate the function of *PoMYBs* in the transcription of genes regulating the progress of primordia, fruiting bodies, and spores.

To identify *PoMYBs* with a possible role in the response to heat stress, we investigated the expression levels of 20 *PoMYBs* in mycelia subjected to high temperatures through RNA-Seq (Figure 4a) and qPCR (Figure 5b). Most *PoMYBs* were induced by heat stress, except *PoMYB01*, *PoMYB08*, *PoMYB09*, and *PoMYB10*. Specially, *PoMYB12* and *PoMYB15* showed extremely high expression levels after heat stress, indicating their important roles in response to heat stress.

## 3. Discussion

This study presents a draft genome of *P. ostreatus*, which is a commercially important representative of oyster mushrooms cultivated in China. The combination of optical mapping and NGS techniques represents an effective approach for de novo whole-genome sequencing, as optical mapping can improve contiguity of assembly and provide a valuable framework for super-scaffolding in the absence of a genetic map. This is the first application of optical mapping in the assembly of a mushroom genome, even if it has been widely used to guide high-confidence assembly in humans [51], plants [52], and animals [53,54]. The assembled 34.76 Mb genome is very close to the other two *P. ostreatus*, CCEF00389 (34.9 Mb) [6] and PC15 (34.34 Mb) [5], but smaller than other sequenced mushrooms, such as *P. tuoliensis* (40.83 Mb) [11], *L. edodes* (41.8 Mb) [15], *V. volvacea* (36.45 Mb) [12], and *Ganoderma lucidum* (43.3 Mb) [55]. Among the three sequenced *P. ostreatus* strains, CCMSSC03989 shows a higher affinity with PC15, and they share 7786 (69.84%) orthologous groups (Figure 2b) and have the highest sequence colinearity (Figure S3). CCMSSC03989 is considered as a strain of strong resistance and high yield [9]. The genome sequences can provide a solid foundation for mining genes of important agronomic traits, such as quality, resistance, and adaptability.

The *MYB* gene family has been found as the largest transcription factor family in plants, and it is involved in numerous signaling pathways. To date, many *MYB* genes have been identified in different plant species. In particular, 198 *MYB* genes were identified in *Arabidopsis* [43], 183 in *O. sativa* subsp. *Indica* [32], 252 in *G. max* [44], 524 in *G. hirsutum* [45], and 458 in *Brassica rapa* ssp. *pekinensis* [46]. In this study, the *MYB* gene family of *P. ostreatus* was analyzed at the genome-wide level*. P. ostreatus* has 20 *MYB* genes, which is more than that of *Magnaporthe oryzae* (13), *Botrytis cinerea* (15), *Ascochyta rabiei* (16), and *Fusarium graminearum* (19) [56], and less than that of *Neurospora crassa* (36) and *Laccaria bicolor* (37) [57]. The size of the *MYB* gene family of *P. ostreatus* is smaller than those of plants, but larger than those of animals, which generally comprise four or five *MYBs* [58]. Previous studies showed that the huge *MYB* gene families in plants are generated due to gene expansion (tandem and segmental duplication) [59,60,61]. In the genome of *P. ostreatus* strain CCMSSC03989, *PoMYB09* was found as a duplication of *PoMYB03*. This may be a recent duplication event, as the same duplication was not observed in that of PC15 and CCEF00389.

The members that fall in a given clade in the phylogenetic analyses may undergo common evolutionary origins and harbor conserved function domains. In the present study, comparative analysis (Figure S4) of *PoMYBs* with other *MYB* genes (plants, fungi, and animals) suggested both conservation and divergence for *PoMYBs*. Twenty *PoMYBs* were clustered into 14 different clades in the phylogenetic tree, indicating that *PoMYBs* is highly divergent within *P. ostreatus* and that most *PoMYBs* are of an ancient origin rather than products of recent gene duplication.

Gene expression patterns can provide important clues for gene function. *MYB* gene families exhibit great disparities in abundance among different organisms and different tissues to exert different physiological functions [60,61]. In this study, *PoMYBs* showed different expression patterns. Certain *PoMYBs* exhibited the highest transcript abundance in a specific stage during reproductive growth. These *PoMYBs* may regulate specific development progress through the regulation of different target genes. A considerable amount of research demonstrated that *MYB* genes are indispensable in spore formation and development [33,38]. In this study, the expression levels of *PoMYB03*, *PoMYB08*, *PoMYB09*, and *PoMYB10* exhibited drastic upregulation (more than 30-fold in qPCR results) in the spores, and thus we speculated that these four genes participate in spore-related processes. Many studies showed that *MYB* genes play important roles in response to abiotic stress [36,37]. The upregulation of the *PoMYBs* under heat stress implied their wide involvement in the heat stress response. The induced transcription of *PoMYBs* regulates various genes [62] to cope with the environmental changes, thereby helping the mycelia to defend themselves against heat stress. Among the 20 *PoMYBs*, *PoMYB02*, *PoMYB12*, *PoMYB13*, *PoMYB16*, *PoMYB17*, and *PoMYB18* exhibited elevated expression during the reproductive stages and under the condition of heat stress, indicating their multifunctional roles in the mushroom development and heat stress response of *P. ostreatus*. In this study, the *PoMYBs* that clustered in the same clade did not show similar expression patterns. One possible reason is that close *PoMYBs* in the phylogenetic tree are similar in MYB domains, but may not be in full-length amino acids or rest domains which may have other functions.

The structural, phylogenetic, and expression analyses of *PoMYBs* provided an insight into the comprehensive functional characterization of the *MYB* gene family for *P. ostreatus*, as well the other mushrooms. The functional exploration of *MYBs* will also facilitate the better understanding of gene regulation in mushrooms by MYB-type transcription factors and marker-assisted breeding in the future.

## 4. Materials and Methods

### 4.1. Strains and Culture Conditions

The dikaryotic *P. ostreatus* strain CCMSSC03989 was used in this study and was maintained on potato dextrose agar (PDA) at 4 °C. For whole genome sequencing, the monokaryotic strain CCMSSC03989-1 was first obtained by dedikaryotization [6] from CCMSSC03989. Then, the vegetative mycelia of CCMSSC03989-1 were collected for DNA isolation after cultivation on PDA in the dark at 28 °C for seven days.

### 4.2. Genome Sequencing, Assembly, and Annotation

Approximately 100 μg of genomic DNA samples were used for genome sequencing on the Illumina Miseq platform. One paired-end (PE) 250 shotgun library (300 bp) and one mate-pair library (5000 bp) were constructed, and 53.73 M raw reads were produced. The reads were filtered with Skewer [63]. Default parameters were used. Finally, 53.72 M reads (Table S1) were retained for genome assembly through AbySS 2.0 [64].

For constructing the optical map, high-molecular-weight DNA was extracted and labeled (endonuclease enzyme Nt.BspQI) following protocols provided by Bionano Genomics, Inc. (San Diego, CA, USA) (https://bionanogenomics.com/support/). The prepared sample was then loaded onto Irys chips (Bionano Genomics, Philadelphia, PA, USA) and then applied to the chip nanochannels. In total, 163,785 raw molecules (Table S2) were produced, and the raw data were filtered by using IrysView 2.4 with default parameters, after which the clean data were assembled by using BionanoSlove (parameters in Supplementary File S1) to construct genome maps.

Hybridscaffolding was performed on the genome map and NGS scaffolds using the Bionano HybridScaffolding pipeline (parameter file is available in the Supplementary File S2) for constructing final scaffolds. Finally, GapCloser was employed to conduct gap closure with shotgun data [65]. KAT [66] and BUSCO v3.0.2 [67] analyses were used to test the completeness of the assembled genome. In addition, the final scaffolds were anchored to 11 chromosomes of the *P. ostreatus* (PC15) genome using the Bionano HybridScaffolding pipeline (Supplementary File S3).

Dispersed repeated sequences of the DNA level were detected through an approach combining de novo prediction and homology-based searching. First, RepeatModeler v1.0.11 (http://www.repeatmasker.org/RepeatModeler/) was used to construct the de novo repeat library, and then the de novo library was mixed with Repbase (a database of eukaryotic repetitive elements) to conduct repeat searching using RepeatMasker v4.06 (http://www.repeatmasker.org/RMDownload.html). The tandem repeats in the genome assembly were identified through the tandem repeat finder TRF v4.0.9 [68].

The gene models of the *P. ostreatus* genome were predicted using the MAKER-P pipeline [69], and further gene function annotation was conducted. First, each protein was searched against the NR [70], Swiss-Prot [71], and InterProscan [72] databases. The best similar hit with an E-value < 10^−5^ was considered to be the gene annotation information. Second, each protein was annotated according to the GO database [73], and Blast2GO was used to obtain GO terms representing a biological process, cellular component, and molecular function. Finally, all proteins were searched against the KEGG database [74] with the KAAS tool [75].

### 4.3. Phylogenetic Analysis and Molecular Clock Estimation

In addition to the *P. ostreatus* genome, we used 33 other fungi genomes for cluster identification to determine the orthologous genes and elucidate the evolution of the genomes (Table S7). The protein sequences of these 34 fungi were compared by using BLASTP with an E-value < 10^−5^ and hit number <500. Then, the BLASTP [76] result was analyzed by using OrthoMCL v2.0.9 [77]. Default parameters were used for acquisition of the orthologous genes, and 131 single-copy orthologous genes were determined. Multiple sequence alignments of these 131 genes were aligned by using MUSCLE 3.8.31 [78] and were combined into a long sequence for each species. Then, the conserved block regions of the alignment were picked out by using Gblocks 0.91b [79] with default parameters, and the final alignment length was 22,649 amino acids. With the input of this alignment, the phylogenetic tree was constructed by using RAxML 8.2.11 [80] with bootstrap 1000. Three fossil calibration points were fixed in the molecule clock analysis [81]: the most recent common ancestor (MRCA) of *Coprinopsis cinerea*, *Laccaria bicolor*, and *Schizophyllum commune* was diverged at 122.74 million years ago (mya); the MRCA of *Serpula lacrymans* and *Coniophora puteana* were diverged at 104.23 mya; the MRCA of *Pichia stipitis*, *Aspergillus niger*, *Cryphonectria parasitica*, *Stagonospora nodorum*, and *Trichoderma reesei* was diverged at 517.55 mya. Then, the divergence time of other nodes was calculated by using r8s v1.81 [82] with TN algorithm, PL method, and the smoothing parameter value was set to 1.8 through cross-validation.

### 4.4. Identification, Cloning, and Classification of the MYB Gene Family in P. ostreatus

*MYB* genes of *P. ostreatus* were identified by using PfamScan (http://www.ebi.ac.uk/Tools/pfa/pfamscan). After manual correction, specific primers (Table S8) were designed for the amplification of the full-length sequences of these *MYB* genes from CCMSSC03989 cDNA. The amplified products were purified and cloned into the pGEM-T vector (Promega, Madison, WI, USA) for sequencing. Finally, 20 *MYB* genes were successfully amplified and sequenced. The MYB-domain was analyzed by the online tool (https://www.ncbi.nlm.nih.gov/Structure/cdd/docs/cdd_search.html), and the *PoMYBs* were classified to 3R-MYB, 2R-MYB, and 1R-MYB based on the repeat unit numbers of MYB-domain.

### 4.5. Sequence Analysis, Gene Structure, and Phylogenetic Analysis of MYB Genes

The pI and molecular weight were computed using the online Compute pI/Mw tool (http://web.expasy.org/compute_pi/). The gene structures of *PoMYB* genes were investigated using in-house script based on the coding sequences and corresponding genomic sequences defined in the gff file. Multiple sequence alignment of these 20 *PoMYB* genes was performed using ClustalOmega [83] based on their MYB domains, and a ML phylogenetic tree was constructed using MEGA 6.0 software [84] with 1000 bootstrap replicates. To further examine the phylogenetic relationship and evolutionary history of the *P. ostreatus MYB* genes, an additional 104 MYB protein sequences (including fungi, plants, and animals) were collected from NCBI (Table S9), and a ML tree based on MYB domains was built using RAxML [80] with 1000 bootstrap replicates. The positions and sequences of MYB domains were in Supplementary File S4.

### 4.6. RNA-Sequencing and Analysis

The dikaryotic strain CCMSSC03989 was used for fruiting-body growth and heat stress treatment as described previously [23]. During fruiting-body growth, four samples were collected, including mycelia, primordia, fruiting bodies, and separated spores. For heat stress treatment, the mycelia were cultured at 40 °C for 48 h with cultures at 28 °C used as controls (the same as mycelia collected during the fruiting-body growth). Three replicates were collected for each sample, and were frozen in liquid nitrogen immediately after collection. In total, five samples including mycelia (28 °C), heat stress-treated mycelia (40 °C), primordia, fruiting bodies, and spores were applied for RNA-Seq and qPCR.

For each sample, three replicates were pooled and then used for RNA extraction. Total RNA was extracted according to the instruction manual of the E.Z.N.A. Plant RNA Kit (Omega Bio-Tek, Norcross, GA, USA). The integrity and concentration of RNA were estimated with an Agilent 2100 Bioanalyzer (Agilent Technologies, Palo Alto, CA, USA) using the Total RNA Nano Kit (RNA 6000 Nano LabChip, Palo Alto, CA, USA). Sequencing libraries were generated using a NEBNext Ultra^TM^ RNA Library Prep Kit for Illumina (New England Biolabs, Ipswich, MA, USA) following the manufacturer’s recommendations. Then, we added index codes to attribute sequences to each sample. The library preparations were sequenced on an Illumina Hiseq 4000 platform and paired-end 150 bp reads were generated. Raw reads generated by RNA-seq were trimmed and quality controlled by Skewer [63] with the following parameters: adapter sequences searching and trimming with 10% max mismatch rate, trimming reads from the 3′ end until Q > 20, and trimmed reads with read length <100 bp or average quality <30 were filtered out. HISAT and StringTie [85] were used to calculate the expression level for each transcript (FPKM). Differentially expressed genes (DEGs) among the different samples were identified by using the edgeR package. The hierarchical clustering analysis of *PoMYB* genes was performed using the pheatmap package in R.

### 4.7. Expression Analysis by qPCR

Total RNA obtained from Section 4.6 was used for qPCR. First-strand cDNA was synthesized with a HiScript II 1st Strand cDNA Synthesis Kit (+gDNA wiper) (Vazyme, Nanjing, China) according to the manufacturer’s instructions. ChamQ Universal SYBR qPCR Master Mix (Vazyme, Nanjing, China) was used for the qPCR as described previously [23]. Glyceraldehyde 3-phosphate dehydrogenase (*gapdh*) was used as reference. Primers were designed by using the DNAMAN software v5.2.2 (Lynnon LLC, San Ramon, CA, USA) (Table S10) and were synthesized by Sangon Biotech Co., Ltd. (Shanghai, China). The relative expression of the genes was calculated with the 2^−ΔΔ*C*t^ method [86]. Gene expression levels in the mycelia were used as reference in the ΔΔ*C*_t_ calculation during the developmental stages, and mycelia cultured at 28 °C were used as references in the heat stress treatment. The means and standard deviations were calculated from the experiments performed in triplicate. Parametric one-way analysis of variance (ANOVA) followed by Duncan’s test and *t*-test were used to calculate significant differences of gene expression of developmental stages (*p* < 0.05) and heat stress, respectively.

### 4.8. Data Availability and Accession Numbers

This whole genome shotgun project has been deposited at DDBJ/ENA/GenBank under the accession number QLNW00000000.

## Figures and Tables

**Table 1:** Statistical analysis of the *P. ostreatus* draft genome.

Contents	Final Scaffolds	Next-Generation Sequencing Assembly	Bionano Genome Map
Number of scaffolds/maps	203	825	246
Total length of scaffolds/maps (Mb)	34.76	36.42	33.69
Scaffold/map N50 (kb)	1109.40	432.52	138
Gap ratio (%)	0.91	3.37	-
Number of contigs	570	1648	-
Total length of contigs (Mb)	34.45	35.19	-
Contig N50 (kb)	191.92	126.35	-
Repeat (%)	10.11	-	-
GC content (%)	50.49	50.90	-
Number of protein-coding genes	10,936	-	-
Average coding gene length (bp)	1743	-	-

## Supplementary Materials

Supplementary materials can be found at http://www.mdpi.com/1422-0067/19/7/2052/s1.

## Abbreviations

NGS	Next-generation sequencing
BUSCO	Benchmarking universal single-copy orthologs
KAT	K-mer analysis toolkit
ML	Maximum likelihood
MRCA	Most recent common ancestor
Mya	Million years ago
RNA-Seq	RNA-sequencing
FPKM	Fragments per kilobase of exon per million mapped reads
qPCR	Quantitative PCR
