# Supplementary material for: Genome-Wide Characterization and Expression Analyses of Pleurotus ostreatus MYB Transcription Factors during Developmental Stages and under Heat Stress Based on de novo Sequenced Genome
Source: Int J Mol Sci. 2018 Jul 14;19(7):2052. doi: 10.3390/ijms19072052 (PMC6073129; doi:10.3390/ijms19072052)
Supplement: Supplementary file 1 [file ijms-19-02052-s001.zip › ijms-325834-supplementary/supplementary/Supplementary Table S1.docx]

**Supplementary Table S1.** Sequencing statistics of *P. ostreatus*.

| **Insert size (bp)** | **Raw Data** | | | **Clean Data** | | |
| --- | --- | --- | --- | --- | --- | --- |
|  | **Total data (Gb)** | **Total read number** | **Sequence depth (X)** | **Total data (Gb)** | **Total read number** | **Sequence depth**  **(X)** |
| 300 | 10.66 | 42,630,096 | 306.87 | 10.51 | 42,617,650 | 302.50 |
| 5,000 | 1.66 | 11,104,876 | 47.85 | 1.35 | 11,102,404 | 38.91 |
| Total | 12.32 | 53,734,972 | 354.72 | 11.86 | 53,720,054 | 341.41 |
