# Supplementary material for: Genome-Wide Characterization and Expression Analyses of Pleurotus ostreatus MYB Transcription Factors during Developmental Stages and under Heat Stress Based on de novo Sequenced Genome
Source: Int J Mol Sci. 2018 Jul 14;19(7):2052. doi: 10.3390/ijms19072052 (PMC6073129; doi:10.3390/ijms19072052)
Supplement: Supplementary file 1 [file ijms-19-02052-s001.zip › ijms-325834-supplementary/supplementary/Supplementary Table S10.docx]

| **Name** | **Forward Sequence(5′–3′)** | **Reverse Sequence (5′–3′)** | **Product Size (bp)** |
| --- | --- | --- | --- |
| *PoMYB01* | ACGGCACCTGCGAGTCTCAA | AGTGGAAGGCGATAGGCTGGAT | 134 |
| *PoMYB02* | CGGATAGGACCAGTGCCAGTGA | TGCTCTCGGCGGTGATTCTGT | 123 |
| *PoMYB03* | CCACCACCACCACCTTCATCTC | TCCAACGCAGCAATGACCTTGA | 221 |
| *PoMYB04* | GGACGGCATTCACGGAACTTCG | GCCACTCCAAGACAGCGGTAGA | 237 |
| *PoMYB05* | GCACCGCAGCAGGACAATGT | TGCCACCTCCAGGACCGATATG | 118 |
| *PoMYB06* | CGCCACAGACATCGCCACAA | AGCAGGGTCAGAGGCAGCAT | 182 |
| *PoMYB07* | AAGCGAATCACAGGCGACTAGC | TGCCGTGCGAGGAGTTACTGA | 155 |
| *PoMYB08* | CCACCACCACCACCTTCATCTC | TCCAACGCAGCAATGACCTTGA | 221 |
| *PoMYB09* | CCACCACCACCACCTTCATCTC | TCCAACGCAGCAATGACCTTGA | 221 |
| *PoMYB10* | ATGGACGGATGCCGATGACAAG | TTGCGACTACCCTTGCTCCTCT | 153 |
| *PoMYB11* | ACCTCCTCCGCAATGCAGTGA | TCAGCCGTTCGTCTTCGTCAGT | 180 |
| *PoMYB12* | ATTGTTCGTCCGTTCGCTACCA | CCAGCCGTCATTCGCAGAGTT | 167 |
| *PoMYB13* | GCGAATCCGATGCTGGTTTGG | ATCCGAAGCCGACTGCGTATC | 263 |
| *PoMYB14* | ACGCCAAGACTCACCTCTCCTC | TCAGTGAACGGTCGTCGCTTCT | 102 |
| *PoMYB15* | GCGGCATACCTGGCAGTCAT | GTCGTTCGTTGGCTCCTCGTTA | 235 |
| *PoMYB16* | GCAGACGGTGGCAGACATTGA | ACCAGATACGGCTTCGGTAGGA | 240 |
| *PoMYB17* | GCCGCCACAACCGTACATAGAA | ACCGCCGTAAGTGCTCTTGATG | 217 |
| *PoMYB18* | GCGGCTTAACGATGCGGACTT | GCACCACGGTAGCGAGGTATTC | 278 |
| *PoMYB19* | GAATAATCGCCGCCGTCCTCTC | GAAACCGTCCCACCGTGAACAA | 270 |
| *PoMYB20* | TCCGAGTCTTACCGCCATTACG | AAGCCGCAGTCTGGACGATAG | 205 |

**Supplementary Table S10.** Primers used in qPCR of *PoMYBs*
