# Supplementary material for: Genome-Wide Characterization and Expression Analyses of Pleurotus ostreatus MYB Transcription Factors during Developmental Stages and under Heat Stress Based on de novo Sequenced Genome
Source: Int J Mol Sci. 2018 Jul 14;19(7):2052. doi: 10.3390/ijms19072052 (PMC6073129; doi:10.3390/ijms19072052)
Supplement: Supplementary file 1 [file ijms-19-02052-s001.zip › ijms-325834-supplementary/supplementary/Supplementary Table S2.docx]

**Supplementary Table S2.** DNA molecule statistics of *P. ostreatus*.

| **Contents** | **Raw molecules** | **Filtered molecules(>150Kb)** |
| --- | --- | --- |
| Number of molecules | 163,785 | 25,001 |
| Total length(Gb) | 21.19 | 7.05 |
| Average length(Kb) | 129.36 | 282.16 |
| Molecule N50(Kb) | 117.9 | 305.7 |
| Label Density(/100Kb) | 10.72 | 10.07 |
