# Supplementary material for: Genome-Wide Characterization and Expression Analyses of Pleurotus ostreatus MYB Transcription Factors during Developmental Stages and under Heat Stress Based on de novo Sequenced Genome
Source: Int J Mol Sci. 2018 Jul 14;19(7):2052. doi: 10.3390/ijms19072052 (PMC6073129; doi:10.3390/ijms19072052)
Supplement: Supplementary file 1 [file ijms-19-02052-s001.zip › ijms-325834-supplementary/supplementary/Supplementary Table S3.docx]

**Supplementary Table S3**. BUSCO analysis of the assembled *P. ostreatus* genome.

| **Contents** | **BUSCOs** | **Ratio (%)** |
| --- | --- | --- |
| Complete | 273 | 94.1 |
| Fragmented | 5 | 1.7 |
| Missing | 12 | 4.2 |
