# Supplementary material for: Genome-Wide Characterization and Expression Analyses of Pleurotus ostreatus MYB Transcription Factors during Developmental Stages and under Heat Stress Based on de novo Sequenced Genome
Source: Int J Mol Sci. 2018 Jul 14;19(7):2052. doi: 10.3390/ijms19072052 (PMC6073129; doi:10.3390/ijms19072052)
Supplement: Supplementary file 1 [file ijms-19-02052-s001.zip › ijms-325834-supplementary/supplementary/Supplementary Table S4.docx]

**Supplementary Table S4**. Statistical analysis of the anchored pseudo chromosomes of *P. ostreatus*.

| **Contents** | **Number/Length** |
| --- | --- |
| Anchored scaffolds | 69 |
| Anchored scaffolds total length (Mb) | 29.48 |
| Anchored scaffolds N50 length (Mb) | 0.727 |
| Anchored scaffolds max length (Mb) | 2.892 |
| Pseudo chromosome (including mitochondrion) | 12 |
| Pseudo chromosome total length (Mb) | 34.453 |
| Pseudo chromosome N50 length (Mb) | 3.269 |
| Pseudo chromosome max length (Mb) | 4.829 |
