# Supplementary material for: Genome-Wide Characterization and Expression Analyses of Pleurotus ostreatus MYB Transcription Factors during Developmental Stages and under Heat Stress Based on de novo Sequenced Genome
Source: Int J Mol Sci. 2018 Jul 14;19(7):2052. doi: 10.3390/ijms19072052 (PMC6073129; doi:10.3390/ijms19072052)
Supplement: Supplementary file 1 [file ijms-19-02052-s001.zip › ijms-325834-supplementary/supplementary/Supplementary Table S5.docx]

**Supplementary Table S5.** Structure variations between PC15 and CCMSSC03989.

| **SV type** | **Count** | **Average length/size change (bp)** |
| --- | --- | --- |
| Deletion | 79 | 8,414.46 |
| Insertion | 56 | 5,400.31 |
| Inversion | 2 | 116,824.25 |
| Total | 137 | - |
