# Supplementary material for: Genome-Wide Characterization and Expression Analyses of Pleurotus ostreatus MYB Transcription Factors during Developmental Stages and under Heat Stress Based on de novo Sequenced Genome
Source: Int J Mol Sci. 2018 Jul 14;19(7):2052. doi: 10.3390/ijms19072052 (PMC6073129; doi:10.3390/ijms19072052)
Supplement: Supplementary file 1 [file ijms-19-02052-s001.zip › ijms-325834-supplementary/supplementary/Supplementary Table S6.docx]

**Supplementary Table S6**. Classification of repeated sequences in *P. ostreatus*.

| **Type** | **Number** | **Total Length (bp)** | **In Genome (%)** |
| --- | --- | --- | --- |
| SINEs | 17 | 3,038 | 0.01 |
| LINEs | 376 | 125,889 | 0.36 |
| LTR elements | 1,941 | 1,486,551 | 4.28 |
| DNA elements | 776 | 346,805 | 1.00 |
| Unclassified | 2,103 | 1,312,743 | 3.78 |
| **Total interspersed repeats** | | **3,275,026** | **9.43** |
| Small RNA | 89 | 25,008 | 0.07 |
| Satellites | 26 | 3,405 | 0.01 |
| Simple repeats | 4,055 | 182,608 | 0.53 |
| Low complexity | 573 | 30,792 | 0.09 |
| **Total repeats** | | **3,516,839** | **10.11** |
