# Supplementary material for: Genome-Wide Characterization and Expression Analyses of Pleurotus ostreatus MYB Transcription Factors during Developmental Stages and under Heat Stress Based on de novo Sequenced Genome
Source: Int J Mol Sci. 2018 Jul 14;19(7):2052. doi: 10.3390/ijms19072052 (PMC6073129; doi:10.3390/ijms19072052)
Supplement: Supplementary file 1 [file ijms-19-02052-s001.zip › ijms-325834-supplementary/supplementary/Supplementary Table S7.docx]

**Supplementary Table S7.** Genome list used for phylogenetic estimation.

| **Species** | **URL** |
| --- | --- |
| *Agaricus bisporus* | <http://genome.jgi.doe.gov/Agabi_varbisH97_2/Agabi_varbisH97_2.home.html> |
| *Aspergillus niger* | <http://genome.jgi.doe.gov/Aspni_DSM_1/Aspni_DSM_1.home.html> |
| *Coprinopsis cinerea* | <http://www.broadinstitute.org/annotation/genome/coprinus_cinereus/MultiHome.html> |
| *Cryptococcus neoformans* | <http://www.broadinstitute.org/annotation/genome/cryptococcus_neoformans/MultiDownloads.html> |
| *Cryphonectria parasitica* | <http://genomeportal.jgi-psf.org/Crypa2/Crypa2.home.html> |
| *Coniophora puteana* | <http://genome.jgi.doe.gov/Conpu1/Conpu1.home.html> |
| *Dichomitus squalens* | <http://genome.jgi.doe.gov/Dicsq1/Dicsq1.home.html> |
| *Ganoderma lucidum* | <http://www.herbalgenomics.org/galu/> |
| *Gymnopus luxurians* | <http://genome.jgi.doe.gov/Gymlu1/Gymlu1.home.html> |
| *Gloeophyllum trabeum* | <http://genome.jgi.doe.gov/Glotr1_1/Glotr1_1.home.html> |
| *Laccaria bicolor* | <http://genome.jgi-psf.org/Lacbi2/Lacbi2.home.html> |
| *Moniliophthora roreri* | <http://www.ncbi.nlm.nih.gov/Traces/wgs/?val=AWSO01> |
| *Neurospora crassa* | <http://www.broadinstitute.org/annotation/genome/neurospora/MultiDownloads.html> |
| *Phanerochaete chrysosporium* | <http://genome.jgi.doe.gov/Phchr2/Phchr2.home.html> |
| *Pleurotus ostreatus_PC15* | <http://genome.jgi-psf.org/PleosPC15_2/PleosPC15_2.home.html> |
| *Postia placenta* | <http://genome.jgi.doe.gov/Pospl1/Pospl1.home.html> |
| *Pichia stipitis* | <http://genome.jgi-psf.org/Picst3/Picst3.home.html> |
| *Saccharomyces cerevisiae* | <http://downloads.yeastgenome.org/sequence/S288C_reference/orf_protein/> |
| *Schizophyllum commune* | <http://genome.jgi-psf.org/Schco3/Schco3.home.html> |
| *Serpula lacrymans* | <http://genome.jgi.doe.gov/SerlaS7_9_2/SerlaS7_9_2.home.html> |
| *Stagonospora nodorum* | <http://genome.jgi.doe.gov/Stano2/Stano2.home.html> |
| *Trichoderma reesei* | <http://genome.jgi-psf.org/Trire2/Trire2.home.html> |
| *Trametes versicolor* | <http://genome.jgi.doe.gov/Trave1/Trave1.home.html> |
| *Ustilago maydis* | <http://www.broadinstitute.org/annotation/genome/ustilago_maydis/Home.html> |
| *Volvariella volvacea* | <http://genome.jgi.doe.gov/Volvo1/Volvo1.home.html> |
| *Pleurotus tuoliensis* | ftp://ftp.ncbi.nlm.nih.gov/genomes/all/GCA/002/847/145/GCA_002847145.1_ASM284714v1/ |
| *Pleurotus eryngii* | ftp://ftp.ncbi.nlm.nih.gov/genomes/all/GCA/001/717/165/GCA_001717165.1_ASM171716v1/ |
| *Pleurotus ostreatus_CCEF00389* | ftp://ftp.ncbi.nlm.nih.gov/genomes/all/GCA/001/956/935/GCA_001956935.1_ASM195693v1 |
| *Pleurotus ostreatus_CCMSSC03989* | - |
| *Ophiocordyceps sinensis* | ftp://ftp.ncbi.nlm.nih.gov/genomes/all/GCA/001/648/815/GCA_001648815.1_ASM164881v1 |
| *Cordyceps militaris* | ftp://ftp.ncbi.nlm.nih.gov/genomes/all/GCF/000/225/605/GCF_000225605.1_CmilitarisCM01_v01/ |
| *Lentinula edodes* | http://legdb.chenlianfu.com/page/download.html |
| *Rhizopus_delemar* | ftp://ftp.ncbi.nlm.nih.gov/genomes/all/GCA/000/149/305/GCA_000149305.1_RO3/ |
| *Flammulina velutipes* | ftp://ftp.ncbi.nlm.nih.gov/genomes/all/GCA/000/633/125/GCA_000633125.1_Fv1.0/ |
