# Supplementary material for: Genome-Wide Characterization and Expression Analyses of Pleurotus ostreatus MYB Transcription Factors during Developmental Stages and under Heat Stress Based on de novo Sequenced Genome
Source: Int J Mol Sci. 2018 Jul 14;19(7):2052. doi: 10.3390/ijms19072052 (PMC6073129; doi:10.3390/ijms19072052)
Supplement: Supplementary file 1 [file ijms-19-02052-s001.zip › ijms-325834-supplementary/supplementary/Supplementary Table S8.docx]

**Supplementary Table S8.** Primers of full length cDNA amplification and sequence characteristics of *PoMYBs*

| **Name** | **Forward Sequence(5′–3′)** | **Reverse Sequence (5′–3′)** | **Product Size (bp)** | **Amino acids** | **Molecular weight (kDa)** | **pI** | **MYB type** | **Accession number** |
| --- | --- | --- | --- | --- | --- | --- | --- | --- |
| *PoMYB01* | ATGAACAAGGATGCAAATAGAG | TTATGATGTCTTGTAGTTTTGTGTG | 1200 | 400 | 43.75 | 5.95 | 2R | MH510313 |
| *PoMYB02* | ATGTCAAAATCAAGTGGGGC | TTATGCAGTAGCTTGAGTAGG | 2406 | 802 | 89.57 | 6.72 | 1R | MH510321 |
| *PoMYB03* | ATGTTGCTCAAGGAGAGCT | CTAGAGAGAAGATAGCTCAG | 708 | 236 | 25.42 | 5.12 | 1R | MH510316 |
| *PoMYB04* | ATGCCGCCCATCATCGAACG | TTACGATCCCTGGCCAGCAC | 3033 | 1011 | 111.94 | 6.26 | 1R | MH510319 |
| *PoMYB05* | ATGGTTCATGAAAAACTTCTGC | CTATCCATCGACAGCATCCATC | 2346 | 782 | 86.4 | 5.52 | 1R | MH510327 |
| *PoMYB06* | ATGGTGGGTGGAACCATTTTC | TTAATCGTCTGTAGGATCAA | 1944 | 648 | 69.66 | 8.63 | 1R | MH510315 |
| *PoMYB07* | ATGCGGACTGAATTGTCT | TCATGCATGAGGTGTTTCG | 1614 | 538 | 61.05 | 6.00 | 3R | MH510309 |
| *PoMYB08* | ATGCCTTCTTCGACGCAATC | CTAGAGAGAAGATAGCTCAG | 951 | 317 | 34.26 | 5.53 | 1R | MH510308 |
| *PoMYB09* | ATGTTGCTCAAGGAGAGCT | CTAGAGAGAAGATAGCTCAG | 708 | 236 | 25.42 | 5.12 | 1R | MH510314 |
| *PoMYB10* | ATGCCTTCTTCGACGCAATC | TCAAGTACGCACTGATTTGAACC | 261 | 87 | 9.45 | 9.03 | 1R | MH510322 |
| *PoMYB11* | ATGATAGAACGCCGATCG | CTAATTCATTTGTCGATTG | 1185 | 395 | 43.07 | 6.15 | 3R | MH510326 |
| *PoMYB12* | ATGGCAAGTCGCGGTGCAAAC | TTAGCTCAGCTGGAGCTTGT | 1329 | 443 | 46.70 | 4.93 | 1R | MH510323 |
| *PoMYB13* | ATGTCGACAATTGCGCCGC | CTAAGAATCGTCGATGTG | 1803 | 601 | 66.88 | 6.65 | 2R | MH510317 |
| *PoMYB14* | ATGTCGACAAACACCGGTGC | TCAGGGCTTTGTCCTCCG | 732 | 244 | 26.61 | 9.96 | 1R | MH510324 |
| *PoMYB15* | ATGGTGAACATACTTGGTC | CTATGCGTTAGGCGGAAAAG | 1443 | 481 | 55.14 | 7.19 | 1R | MH510318 |
| *PoMYB16* | ATGACTGTAACACATCGG | TTAGTGGGGAGTCCACGCT | 1869 | 623 | 69.48 | 6.12 | 1R | MH510310 |
| *PoMYB17* | ATGGCACCTGCTCGGAGGC | TCAGGCGAGGGCAGCGGCCAT | 2064 | 688 | 77.63 | 6.59 | 3R | MH510325 |
| *PoMYB18* | ATGCCCATCTTCGAGGGCGA | TCAAAGAAGTACAGGGCTTG | 729 | 243 | 27.43 | 8.92 | 1R | MH510320 |
| *PoMYB19* | ATGGTACATAATGGTGAAG | TTAATCTCTCCCTTTCAAC | 888 | 296 | 34.16 | 10.15 | 1R | MH510312 |
| *PoMYB20* | ATGACTGAAAGGAATGTCGG | CTACTTCGCTGATTGTGCTG | 1950 | 650 | 71.42 | 6.43 | 2R | MH510311 |
