# Supplementary material for: Genome-Wide Characterization and Expression Analyses of Pleurotus ostreatus MYB Transcription Factors during Developmental Stages and under Heat Stress Based on de novo Sequenced Genome
Source: Int J Mol Sci. 2018 Jul 14;19(7):2052. doi: 10.3390/ijms19072052 (PMC6073129; doi:10.3390/ijms19072052)
Supplement: Supplementary file 1 [file ijms-19-02052-s001.zip › ijms-325834-supplementary/supplementary/Supplementary Table S9.docx]

**Supplementary Table S9.** MYB proteins used in the phylogenetic study.

| **Accession number** | **Species** | **Accession number** | **Species** | **Accession number** | **Species** |
| --- | --- | --- | --- | --- | --- |
| EAW10221.1 | *Aspergillus clavatus* | EUC62021.1 | *Rhizoctonia solani* | KXH25897.1 | *Colletotrichum salicis* |
| EAW25371.1 | *Aspergillus fischeri* | EXF80140.1 | *Colletotrichum fioriniae* | KXN82675.1 | *Leucoagaricus sp.* |
| ABN67829.2 | *Scheffersomyces stipitis* | KDN69128.1 | *Colletotrichum sublineola* | KXN83638.1 | *Leucoagaricus sp.* |
| XP_001901281.1 | *Brugia malayi* | KEH41857.1 | *Medicago truncatula* | KXX78556.1 | *Madurella mycetomatis* |
| EEB06858.1 | *Schizosaccharomyces japonicus* | KEP53092.1 | *Rhizoctonia solani* | KXX81397.1 | *Madurella mycetomatis* |
| EED15261.1 | *Talaromyces stipitatus* | XP_008649480.1 | *Zea mays* | KYQ41466.1 | *Hypsizygus marmoreus* |
| EED53852.1 | *Aspergillus flavus* | KFX41982.1 | *Talaromyces marneffei* | KYQ45774.1 | *Hypsizygus marmoreus* |
| EER24168.1 | *Coccidioides posadasii* | CEF96818.1 | *Ostreococcus tauri* | KZL71301.1 | *Colletotrichum tofieldiae* |
| CBF70441.1 | *Aspergillus nidulans* | KGQ84339.1 | *Candida albicans* | OAA75458.1 | *Cordyceps confragosa* |
| EFA74690.1 | *Heterostelium album* | KGQ86225.1 | *Candida albicans* | OBZ65830.1 | *Grifola frondosa* |
| EFQ30686.1 | *Colletotrichum graminicola* | KGQ88588.1 | *Candida albicans* | OBZ70249.1 | *Grifola frondosa* |
| EGG13494.1 | *Cavenderia fasciculata* | KGR05094.1 | *Candida albicans* | OBZ79994.1 | *Grifola frondosa* |
| CCD56252.1 | *Botrytis cinerea* | KGR06487.1 | *Candida albicans* | OEL35965.1 | *Dichanthelium oligosanthes* |
| AEV91160.1 | *Triticum aestivum* | KHJ39960.1 | *Trichuris suis* | OGM47090.1 | *Aspergillus bombycis* |
| EKD21697.1 | *Marssonina brunnea* | XP_011081986.1 | *Sesamum indicum* | CZT45444.1 | *Rhynchosporium secalis* |
| ELR13667.1 | *Acanthamoeba castellanii* | KIH59320.1 | *Ancylostoma duodenale* | OJD37353.1 | *Diplodia corticola* |
| ELU38782.1 | *Rhizoctonia solani* | CED83835.1 | *Xanthophyllomyces dendrorhous* | OJT09598.1 | *Trametes pubescens* |
| ELU43958.1 | *Rhizoctonia solani* | CDZ97617.1 | *Xanthophyllomyces dendrorhous* | OKY67416.1 | *Phlebia centrifuga* |
| EMR64234.1 | *Eutypa lata* | CDZ98477.1 | *Xanthophyllomyces dendrorhous* | OKY68124.1 | *Phlebia centrifuga* |
| EOD46023.1 | *Neofusicoccum parvum* | KNG84124.1 | *Aspergillus nomius* | OLL23066.1 | *Neolecta irregularis* |
| XP_004960645.1 | *Setaria italica* | KNZ77566.1 | *Termitomyces sp.* | GAV86755.1 | *Cephalotus follicularis* |
| EPX70666.1 | *Schizosaccharomyces octosporus* | KNZ79637.1 | *Termitomyces sp.* | GAV73735.1 | *Cephalotus follicularis* |
| EPY51827.1 | *Schizosaccharomyces cryophilus* | KOC08701.1 | *Aspergillus flavus* | GAV59866.1 | *Cephalotus follicularis* |
| ESK89543.1 | *Moniliophthora roreri* | CUA70257.1 | *Rhizoctonia solani* | GAV57001.1 | *Cephalotus follicularis* |
| ESK95523.1 | *Moniliophthora roreri* | KVH91680.1 | *Cynara cardunculus* | OMJ19888.1 | *Smittium culicis* |
| GAV99335.1 | *Lentinula edodes* | AQK84939.1 | *Zea mays* | XP_020093195.1 | *Ananas comosus* |
| OQU98109.1 | *Cladophialophora immunda* | OTG10250.1 | *Helianthus annuus* | XP_021613484.1 | *Manihot esculenta* |
| SLM36928.1 | *Umbilicaria pustulata* | OVA12594.1 | *Macleaya cordata* | PBP23513.1 | *Diplocarpon rosae* |
| OTA04419.1 | *Trichoderma parareesei* | OVA18483.1 | *Macleaya cordata* | XP_022750340.1 | *Durio zibethinus* |
| OPB38518.1 | *Trichoderma guizhouense* | OPB42296.1 | *Trichoderma guizhouense* | OPB43485.1 | *Trichoderma guizhouense* |
| AQK84928.1 | *Zea mays* | AQK84941.1 | *Zea mays* | XP_020093199.1 | *Ananas comosus* |
| AQK84929.1 | *Zea mays* | AQK91548.1 | *Zea mays* | OON17689.1 | *Opisthorchis viverrini* |
| AQK84933.1 | *Zea mays* | XP_020103058.1 | *Ananas comosus* | XP_020174664.1 | *Aegilops tauschii* |
| XP_020524755.1 | *Amborella trichopoda* | PIB00133.1 | *Cercospora beticola* | GAW07194.1 | *Lentinula edodes* |
| ETN76391.1 | *Necator americanus* | KXG21588.1 | *Sorghum bicolor* |  |  |
