# Supplementary figures and images for: Genome-Wide Characterization and Expression Analyses of Pleurotus ostreatus MYB Transcription Factors during Developmental Stages and under Heat Stress Based on de novo Sequenced Genome
Source: Int J Mol Sci. 2018 Jul 14;19(7):2052. doi: 10.3390/ijms19072052 (PMC6073129; doi:10.3390/ijms19072052)

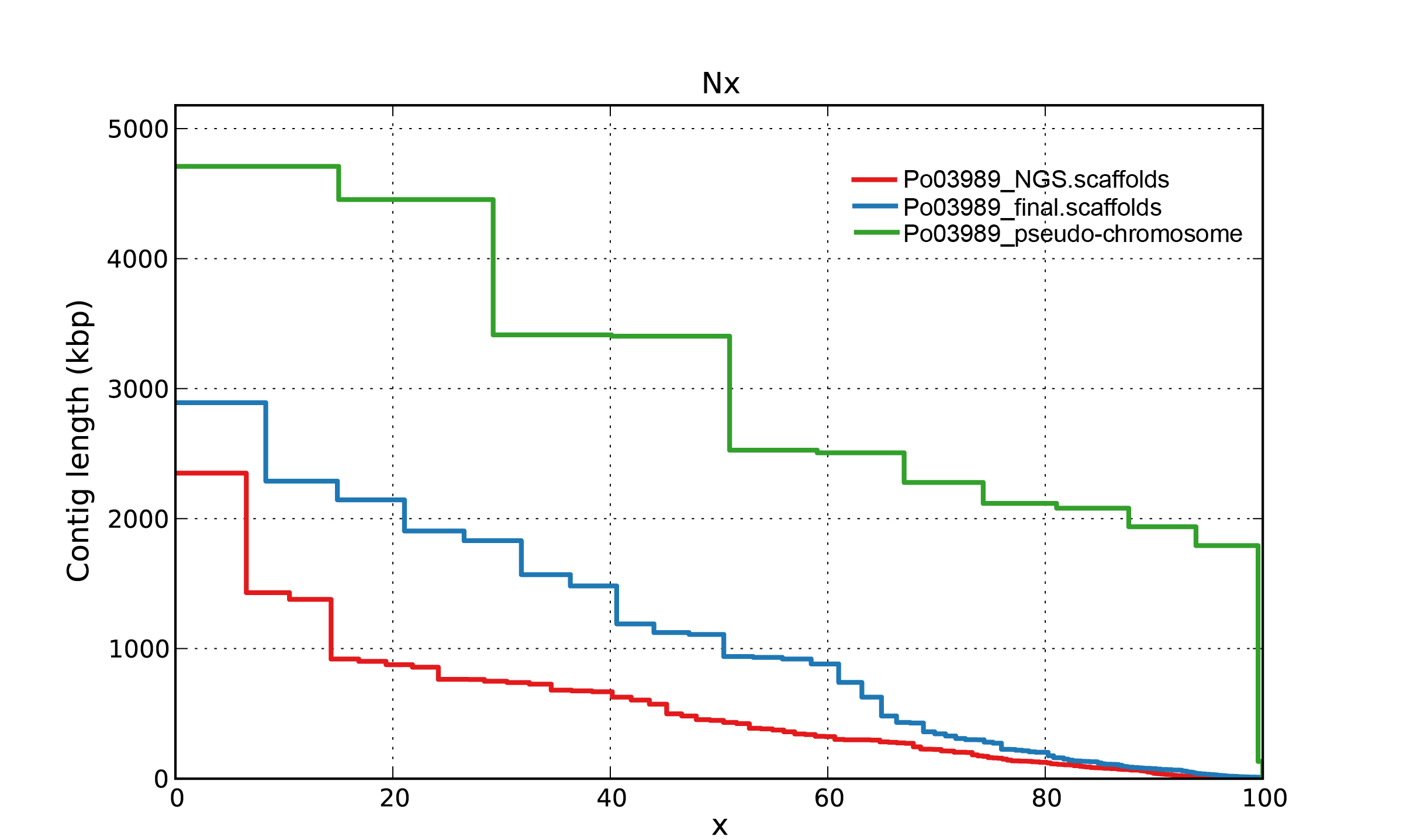

Supplement: Supplementary file 1 [file ijms-19-02052-s001.zip › ijms-325834-supplementary/supplementary/Supplementary Figure S1.jpg]

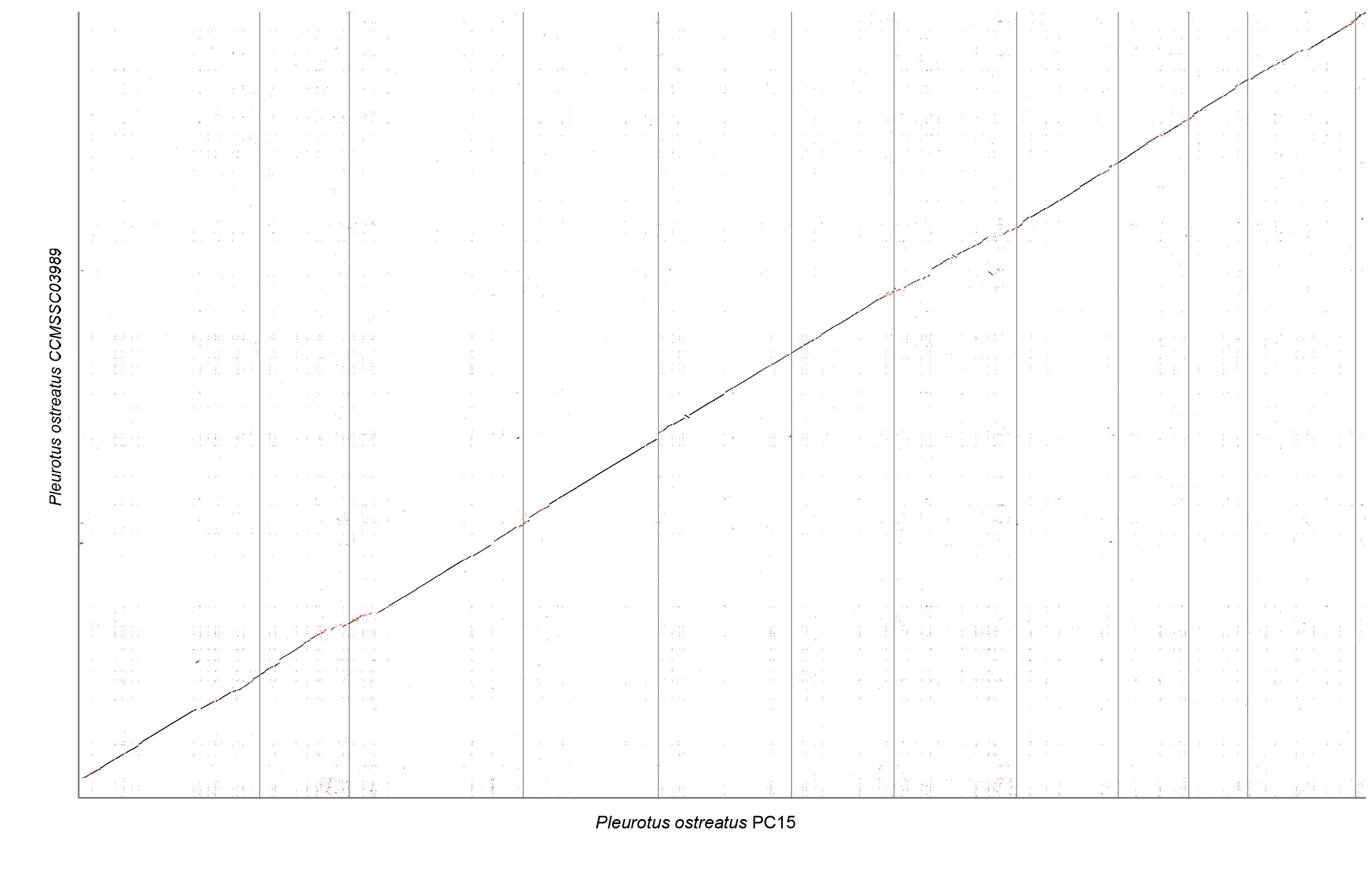

Supplement: Supplementary file 1 [file ijms-19-02052-s001.zip › ijms-325834-supplementary/supplementary/Supplementary Figure S3.jpg]

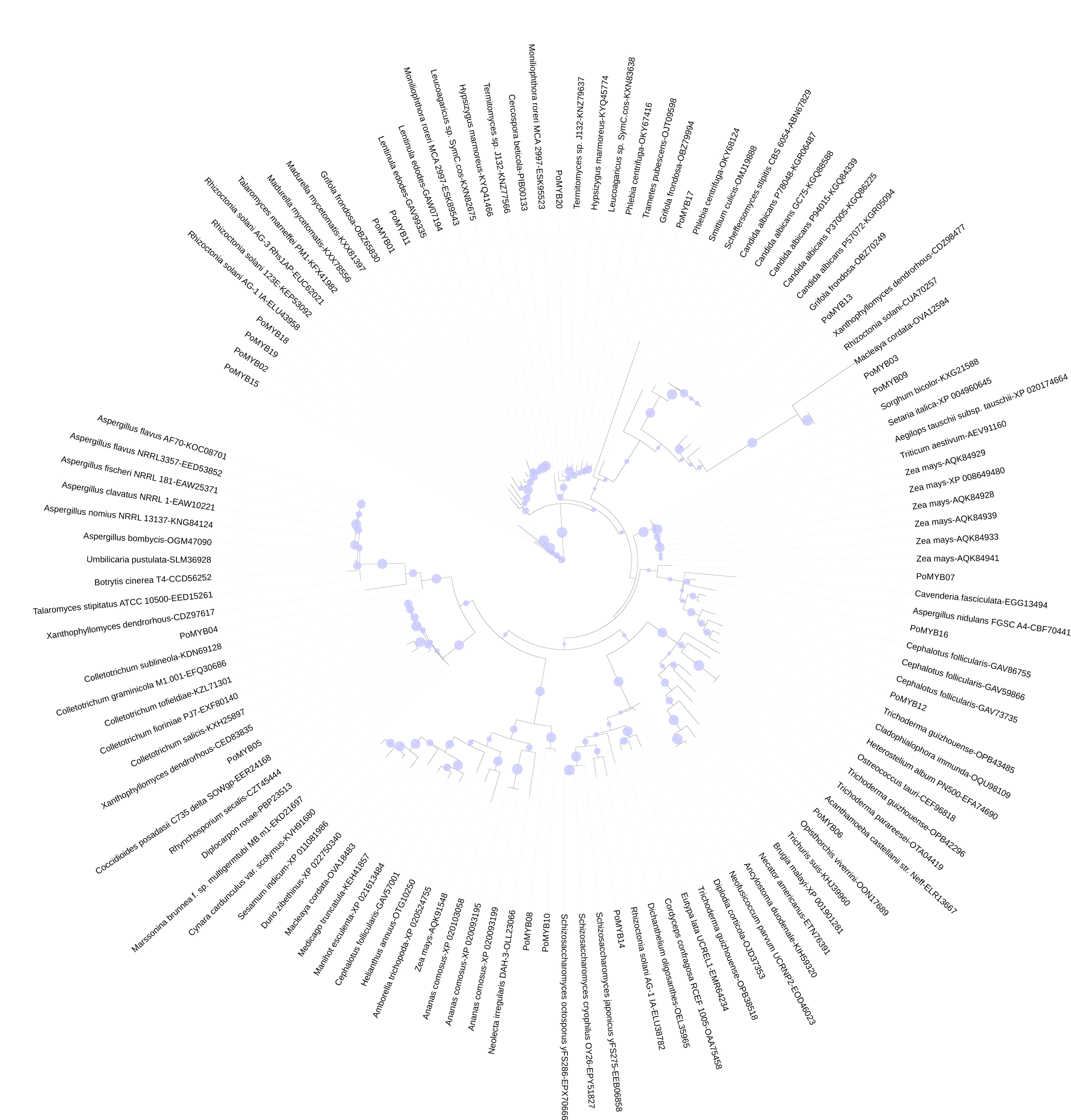

Supplement: Supplementary file 1 [file ijms-19-02052-s001.zip › ijms-325834-supplementary/supplementary/Supplementary Figure S4.jpg]
